# Sex-specific differences in *Juniperus communis*: essential oil yield, growth-defence conflict and population sex ratio

**DOI:** 10.1093/aobpla/plab021

**Published:** 2021-04-22

**Authors:** Gábor Markó, István Németh, Veronika Gyuricza, Vilmos Altbäcker

**Affiliations:** 1 Department of Plant Pathology, Institute of Plant Protection, Hungarian University of Agriculture and Life Sciences, Ménesi út 44, H-1118 Budapest, Hungary; 2 Behavioural Ecology Group, Department of Systematic Zoology and Ecology, Eötvös Loránd University, Pázmány Péter sétány 1/C, H-1117 Budapest, Hungary; 3 Biotech Biostatistics and Programming, Parexel International, Hermina út 17, H-1146 Budapest, Hungary; 4 Department of Ethology, Institute of Biology, Eötvös Loránd University, Pázmány Péter sétány 1/C, H-1117 Budapest, Hungary; 5 Department of Nature Conservation, Institute of Game Management and Nature Protection, Hungarian University of Agriculture and Life Sciences, Guba Sándor utca 40, H-7400 Kaposvár, Hungary

**Keywords:** Chemical defence, dioecy, plant secondary metabolites, resource acquisition, sexual dimorphism, terpenes

## Abstract

In plants, biomass and nutrient allocation often generate trade-offs between the different biochemical pathways conflicting the utilization of the common source among growth, reproduction and chemical defence. However, in dioecious plant species, these trade-off patterns could appear as a more contrasted problem between males and females due to the dissimilar reproduction investment. Generally, the growth ratio is higher in males than females, while females have a stronger defence than males. To understand the possible role of the sex-specific dissimilarities within the growth-defence conflict framework, we investigated the possible causes of the high variance of the essential oil yield in a dioecious evergreen species, *Juniperus communis*. Specifically, we tested the correlations between the essential oil yield with other individual-specific traits (e.g. sex, age), the presence of the growth-defence trade-off, and the differential growth and survival patterns between males and females through an extensive field survey with sample collection in three natural populations (Kiskunság National Park, Hungary). The individual-specific essential oil yield was also measured and served as a proxy to describe the degree of chemical defence. We found that the essential oil yield showed strong and consistent sex-specific patterns decreasing with age in adults. Contrary to the predictions, the males showed a consistently higher yield than the females. We also observed a growth-defence trade-off in males but not in females. Consistently with the growth-defence conflict hypothesis, the populations’ sex ratio was male-biased, and this pattern was more evident with ageing modifying the demographic structure due to the sexually dissimilar lifespan. Our juniper study revealed a contrasting and unique essential oil accumulation driven by the complex allocation trade-off mechanisms within individuals, which could be a flexible and adaptive defence response against the increasing biotic and abiotic environmental stresses exacerbated under global climate change.

## Teaser

Plants often face challenges in sharing nutrient resources among growth, reproduction and chemical defence, especially in dioecious species due to the dissimilar reproduction investment between males and females. This study provides a unique insight into the growth-defence resource allocation conflicts in a less studied and long-lived dioecious conifer species, the common juniper (*Juniperus communis*). We observed sex-specific differences in growth and essential oil accumulation due to conflict between growth and chemical defence, resulting in male-biased populations. We conclude that dioecious plants can respond adaptively to environmental challenges via flexible growth-defence mechanisms.

## Introduction

The plant nutrient allocation trade-offs have been central in both the growth and chemical defence theories (e.g. Freeland and Janzen 1974; Bryant *et al.* 1985; Coley *et al.* 1985; Herms and Mattson 1992; Stamp 2003). Plant growth theories primarily emphasize the nutrient allocation into the functional traits to acquire other nutrient resources (e.g. Ingestad and Göran 1992), while plant defence theories focus on the production of plant secondary metabolites (PSMs), which ensure a highly beneficial chemical-based tissue protection against natural enemies (e.g. Bryant *et al.* 1983; Berenbaum 1995; Stamp 2003). The chemical defence hypothesis predicts that chemical protection should negatively influence other basic metabolic processes (i.e. growth, reproduction) through allocation trade-offs manifesting a physiological cost if nutrient resources are limited (Stamp and Bowers 2000; Koricheva 2002; Züst and Agrawal 2017; Wink 2010).

The nutrient allocation trade-off conflicts can manifest in negative correlations between functionally different phenotypic and genetic traits responsible for defence, growth and reproduction, reflecting the limited allocation of nutrient resources (Koricheva 2002; Züst and Agrawal 2017). Koricheva (2002) found an overall strong negative correlation between defence and fitness traits in woody species; however, the correlation patterns of deciduous and evergreen taxa (*Salix* and *Pinus* dominant, respectively) showed considerable differences (i.e. weak and no trade-offs, respectively) due to the high heterogeneity within evergreens. Similarly, the growth-defence conflicts can also generate inconsistent species-specific allocation differences (Defossez *et al.* 2018), suggesting complex and indirect physiological interferences between different phenotypic and genotypic traits. Moreover, individual differences in both cost–benefit ratio in allocation patterns and stress tolerance can also determine different fitness outputs (e.g. survival) and implicate consequences for ecology (e.g. sex ratio) and evolution (e.g. dioecy) (Ågren *et al.* 1999; Dorken and Van Drunen 2018).

Dioecious plants provide a unique opportunity to test the relative importance of the sex-specific resource allocation differences in growth-defence trade-offs among individuals due to the dissimilar reproduction investments (Ågren *et al.* 1999; Avila-Sakar and Romanow 2012). Studies in dioecious species showed powerful sex-specific effects among phenotypic traits, for example, on growth rate (Matsushita *et al.* 2016) and reproduction cost (Rocheleau and Houle 2001; Queenborough *et al.* 2007). The bias in reproduction investments among sexes could explain these sex-specific phenotypic variations (Coley *et al.* 1985; Jing and Coley 1990; Sutherland 2004). A dioecious female plant invests more in reproduction than a male because, despite both males and females produce reproductive organs with gametes, a female also has to produce fruits nourishing seeds with zygotes inside (Lloyd and Webb 1977; Ortiz *et al.* 2002).

Based on the dissimilar reproduction investment, the growth-defence trade-off predicts that males invest more in compensatory growth than females, while females allocate more into chemical defence to protect generative organs than males (Bañuelos *et al.* 2004; Cornelissen and Stiling 2005). Some studies supported the general pattern underlying sex-specific growth-defence trade-offs along various taxa (Jing and Coley 1990; Elmqvist *et al.* 1991; Cornelissen and Stiling 2005; Jiang *et al.* 2016). Inversely, different sex-specific allocation patterns, in which females tend to invest less into the chemical defence and more into biomass production than males, were also found, where the increased reproductive investment corresponded with a reduced chemical defence level in females (Massei *et al.* 2006; Ward 2007; Litto *et al.* 2015). Nevertheless, other studies without finding sex-specific differences suggested that these trade-off conflicts could be less critical or effectively depressed by other environmental stressors in specific dioecious species (McKown *et al.* 2017; Stark and Martz 2018).

The sex-specific allocation trade-offs can also generate a biased sex ratio on a longer temporal scale (Cornelissen and Stiling 2005; Avila-Sakar and Romanow 2012). The sex with relative lower maintenance or higher defence predicted to be less exposed to herbivores or environmental stressors, promoting higher survival (Field *et al.* 2013a). Examples in dioecious species generally interpreted a male-biased population structure directly linked to different reproduction cost (Falinski 1980; Rocheleau and Houle 2001; Ortiz *et al.* 2002; Matsushita *et al.* 2016). However, the related evidence was often controversial (Marion and Houle 1996; Queenborough *et al.* 2007). Furthermore, within the same dioecious reproduction system, the taxonomic differences in allocation trade-offs could also be the source of such heterogeneity due to the taxon-specific physiological pathways in PSM production modified by the plants’ physiological constraints and other environmental stressors (Züst and Agrawal 2017). Dioecious studies related to the sex-specific chemical defensive traits often paid a closer attention to the phenolics-producer species (e.g. *Salix*, *Populus*), while the terpene-producers (including evergreens) were mostly underrepresented (e.g. Cornelissen and Stiling 2005). However, the mostly monoecious pines have been frequent models to reveal defensive mechanisms; the exceptions (e.g. junipers) can represent the dioecious evergreens and fill the underlying knowledge gaps.

Despite recognizing the scientific potential in juniper as a model, only a few previous studies tested the underlying allocation of trade-offs and their ecological consequences. Therefore, the results are still ambiguous and challenging to interpret (Marion and Houle 1996; Ortiz *et al.* 2002; Massei *et al.* 2006; Ward 2007; Stark and Martz 2018). The common juniper (*Juniperus communis*) is an evergreen, dioecious conifer shrub (Thomas *et al.* 2007), widely distributed in the Northern Hemisphere (Adams *et al.* 2003) occurring across various environmental gradients, including also the most extreme habitats (Kertész *et al.* 1993; García *et al.* 1999; Ortiz *et al.* 2002; Stark and Martz 2018). The common juniper is an extremely slow-growing species with a long lifespan (Grubb *et al.* 1999; Ward 1982, 2007). Long-lived plants occurring in low-source or extreme ecosystems tend to invest more in chemical defence than growth because of the high risk of exposition to biotic and abiotic stressors during their lifespan (Vázquez-González *et al.* 2020). Besides, female cones need 2 or 3 years to ripen (Thomas *et al.* 2007). Simultaneously, junipers provide an essential food source for herbivores (Mátrai *et al.* 1998; Provenza *et al.* 2003; Thomas *et al.* 2007), representing an unusually high selection force (Markó *et al.* 2008; Graora *et al.* 2010). Thus, the characteristics of these species as well as their environmental conditions warrant maintaining an appropriate chemical defence that could be crucial for their survival.

In junipers, similarly to other pines, the chemical defence is primarily based on terpenoids, represented mostly by essential oils, one of the most diverse chemical groups for molecular structure and biochemical function and the accumulation of which represents high metabolic cost (Gershenzon 1994). The essential oil yield can be a good proxy to describe the actual degree of chemical defence due to its high flexibility giving adaptive phenotypic response against specific abiotic and biotic ecological challenges (e.g. Phillips and Croteau 1999).

A juniper model, due to the species-specific features described earlier, could expectedly draw more evident sex-specific patterns in the growth-defence trade-off than the other, less slow-growing conifers (e.g. *Pinus*) or other fast-growing woody taxa (e.g. *Salix*, *Populus*) (Coley *et al.* 1985). We, therefore, investigated the allocation patterns generated by sexual dissimilarities within the growth-defence conflict framework using a complex multilevel approach in this less studied and long-lived dioecious conifer species, *J. communis*. We aimed to (i) understand the role of sex differences affecting the variation of the essential oil yield with covariates (e.g. age, shrub size, canopy position); (ii) test the presence of the growth-defence conflict described by the correlation between the essential oil yield and age-corrected shrub size; (iii) detect differential demographic structure among sexes causing an unbalanced sex ratio within the populations. If the growth-defence conflict plays an essential role in junipers, we would expect sex-specific contrasts in the essential oil yield, the growth, or both. If such trade-off was detected between phenotypic traits, we predicted that juniper males would invest more in growth than females, while females allocate more into the PSMs production than males. We also expected contrasting survival rates as a long-term consequence of the different equilibria in the growth-defence conflicts.

## Materials and Methods

### Habitats and measuring morphology

We performed the field survey in three different juniper populations in the Kiskunság National Park, Hungary (see Supporting Information—Fig. S1). We labelled the population according to the closest village, namely Orgovány (46°47′28″N; 19°27′14″E), Bugac (46°39′11″N; 19°36′18″E) and Bócsa (46°38′56,99″N; 19°27′23,86″E) (details in Kertész *et al.* 1993; Markó *et al.* 2008).

In each population, we randomly selected juvenile (*N* = 10-10, height: 0.25–1 m) and adult juniper shrubs (*N* = 20-20, height: 1–5 m) to quantify their morphology and for performing chemical analyses based on previous juniper studies (García *et al.* 1999; Markó *et al.* 2008) (Table 1). We measured the shrubs’ height by a foot rule (expressed in metre with decimal accuracy) and the basal steam diameter by a calliper (expressed in centimetre with decimal accuracy). For a better biological interpretation, we calculated the age of shrubs using the following formula:

**Table 1.** Summary of the most relevant descriptive information of the measured *Juniperus communis* populations sampled for measuring the yield of essential oil. The table summarized the most relevant descriptive information of the studied *Juniperus communis* populations. Columns contain the sampling sites, age classes, sample sizes (*N*), mean of the age and height, including their standard error (SE) values, while rows contain values by the sampling sites and age categories.

Site	Age class	*N*	Age (year, mean ± SD)	Height (m, mean ± SD)
Bugac	Juvenile	10	7.5 ± 2.7	0.82 ± 0.3
	Adult	20	41.3 ± 11.6	3.68 ± 1.1
Bócsa	Juvenile	10	7.7 ± 2.0	0.95 ± 0.2
	Adult	20	36.8 ± 11.9	4.24 ± 1.2
Orgovány	Juvenile	10	7.1 ± 2.4	0.80 ± 0.3
	Adult	20	41.1 ± 11.2	3.60 ± 1.1


$$\begin{array}{l} y=1.7753+3.8245x, \end{array}$$

where the *x* means the basal steam diameter in centimetre and the *y* represents the age in years. During the field survey, we also identified the sex of the shrubs based on their reproductive organs (female: berries, male: polliniferous flowers) by simple visual observation. We classified the small young shrubs without reproductive organs as infertile juveniles.

### Essential oil yield: sampling and chemical analysis

After measuring the plant morphology, plant samples were collected for quantifying the essential oil yield. In adult shrubs, terminal, the 10–15 cm long terminal twigs with leaves were cut from three different vertical canopy positions (lower: 0–0.5 m; middle: 0.5–1.2 m; upper: 1.2–2 m) from each shrub for testing the within-individual variation in the essential oil yield. As juvenile shrubs had small canopy biomass, only one sample from each was collected, and samples from the entire canopy were included. As juniper berries also contain essential oils, they were removed from the female samples to compare the sexes. The infestation prevalence of juniper scale insect (*Carulaspis juniperi*) was also determined in each collected sample (yes or no). After sampling the shrubs in the field, samples were labelled individually (around 100–120 g each) and transported to the laboratory in a mobile cooler box (5 °C).

In both the sample preparations and the essential oil yield extraction, the quality and analytical standards were strictly followed ( Végh 1986; Markó *et al.* 2008). Immediately after the transportation samples were air-dried (23 ± 3 °C), and stored in a dark, dry and adequately ventilated storage (20 ± 3 °C) until further manipulation. In each sample, the air-dried plant material were chopped into 2 cm pieces, and two subsamples were created to measure both the total yield of essential oil (40–50 g) and the moisture content (4–5 g), separately. The essential oil extraction was performed by a Clevenger-type circulatory hydrodistillation apparatus, including a built-in graduated tube for the volumetric determination of the essential oils at the end of the process (Végh 1986; Baser and Buchbauer 2015). The extracted total essential oil yield was expressed in mL (to 0.0025 mL accuracy) converted to a standard unit (i.e. 100 g dry material) based on the weight and the moisture content of the given sample. For calculating the absolute dry weight of a sample, an oven-drying method was used for measuring the moisture content drying the plant material at 105 ± 0.5 °C in a Memmert U-type oven until reaching the constant mass (3 h).

### Population patterns: growth and sex ratio

For testing the sexual differences in the growth pattern in junipers, a correlative study approach was used to describe the age–height relationship. Firstly, juniper shrubs were randomly selected based on the orthophoto of each population. Secondly, the sex was identified, and the height and the stem diameter measured in each selected individual. The age estimation was the same as mentioned earlier. Note that this dataset partially overlaps with the one used for other research purposes in our previous study (describe age distribution, Markó *et al.* 2008). In the present study, the statistical analyses included only those shrubs whose sexual identification was known (sample sizes: Bugac: *N* = 476, Bócsa: *N* = 368, Orgovány: *N* = 619).

### Statistical analyses

All statistical analyses were performed in the R environment (version: 3.6.2, R Development Core Team 2019).

#### Variation in the essential oil yields.

First, the sex-specific effects on the variation of essential oil yield were analysed, including only the adult individuals by a Linear Mixed-Effect Model (LMM) applied to the ‘*lme4*’ package. The following model structure was used for the statistical analyses: the essential oil yield was entered as a response variable, the focal predictors as factors were the sex, the canopy position, the infestation by scale insect, while the age was presented as a covariate. The model also included the interactions such as the infestation × canopy position, sex × infestation, sex × canopy position, sex × age, infestation × age and canopy position × age. For avoiding the violation related to the standard assumption of independent measurements, the individual identity (ID) was entered as a random factor in a hierarchical structure (ID nested in sex nested in site) due to the repetitive sampling of the same shrubs (i.e. three canopy positions) and to accomplish a statistical control to the random sex- and site-specific effects. A square root transformation was used for the essential oil yield in the final statistical model to achieve the normal distribution of the model residuals.

Second, a separate model was built for testing the age effect on a more extended lifetime scale, including both the young and adult shrubs by a Nonlinear Least Squares Regression (NLS) using the basic ‘*stats*’ package. The model included the individual means of the essential oil yield, a common ground for young and adult shrubs, as the dependent variable, while the age was the predictor variable.

#### Sex-specific differences in growth and growth-defence trade-off.

The plant growth patterns usually show a robust logistic relationship between biomass accumulation and age, a similar association between the size and the age was described in junipers by an NLS logistic regression model applied with SSlogis function. The size was a dependent variable in that model, while the age was the predictor after a logarithmic transformation. Afterwards, the model residuals from the NLS regression model were calculated to test the possible sex-specific effects on the growth of common junipers. Finally, an LMM was run in which the dependent variable was the age-corrected size (residuals), the predictor variable was only the sex as a factor, while the random factor was the site as statistical control to the site-specific effects.

For testing the presence of the growth-defence trade-off, another LMM was run in which the predicted variable was the essential oil yield, while the predictors were the age-corrected size residuals, the sex and their interactions and the random factor was the shrub ID.

#### Population sex ratio.

The Generalized Linear Mixed-Effect Model (using ‘*lme4*’ package) was used for testing the presence of the male-biased sex ratio characterizing the demographic change along with ageing. In this model, the dependent variable was the individual sex as a binomial variable (male or female), while the predictor variables were the site as a factor, the age as a covariate, and their interaction. The identification number of the sampling plots (sampling site within a population) was entered as a random factor. The model fit was carried out by Laplace approximation. The predictors were tested by the maximum-likelihood ratio test implemented by the statistical comparison of the full and the reduced model.

## Results

### Variation in the essential oil yields

**Figure 1.**
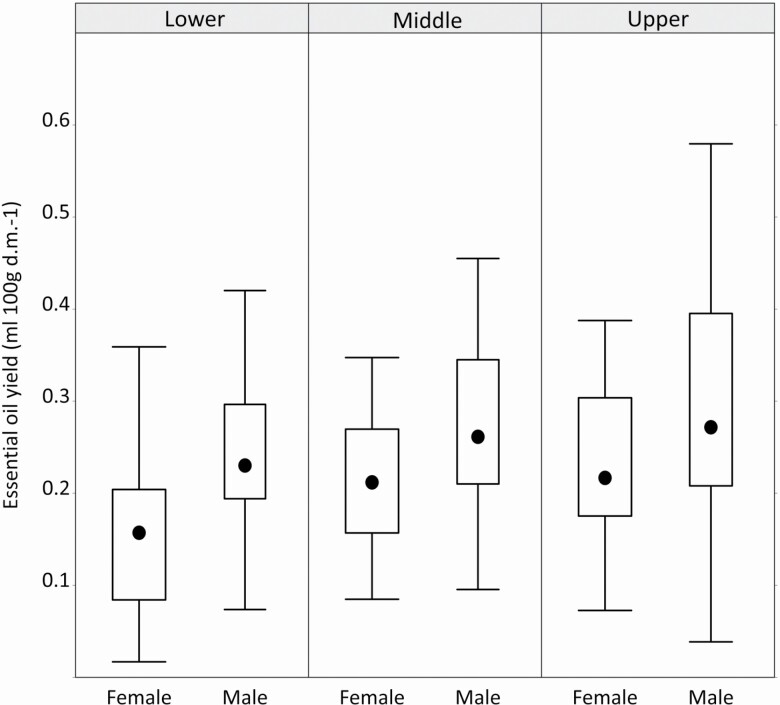
The Box-and-Whisker plot shows the essential oil yield values (expressed in mL/100 g dry mass) based on the canopy positions in juniper males and females, separately. Variability is represented by the medians (black dot), interquartiles (boxes) and interquartile ranges (whiskers).

**Figure 2.**
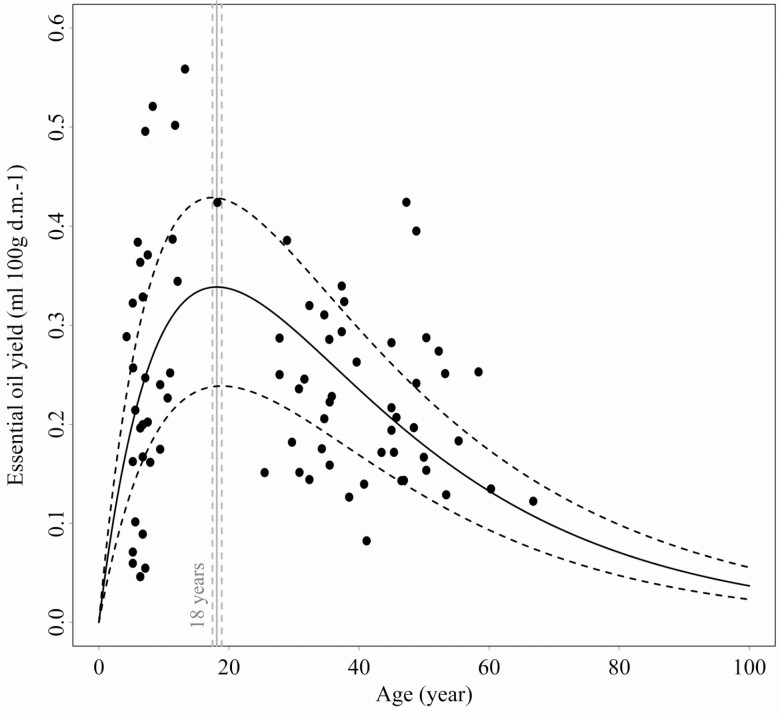
The scatter plot shows a bi-exponential function describing the relationship between essential oil yield (expressed in mL/100 g dry mass) and age in *Juniperus communis*. The estimated function (continuous black line) was fitted with 95 % confidence intervals (dashed black lines). The estimated maximum peak of the essential oil yield was also demonstrated (continuous vertical grey line) with 95 % confidence intervals (dashed vertical grey lines).

**Figure 3.**
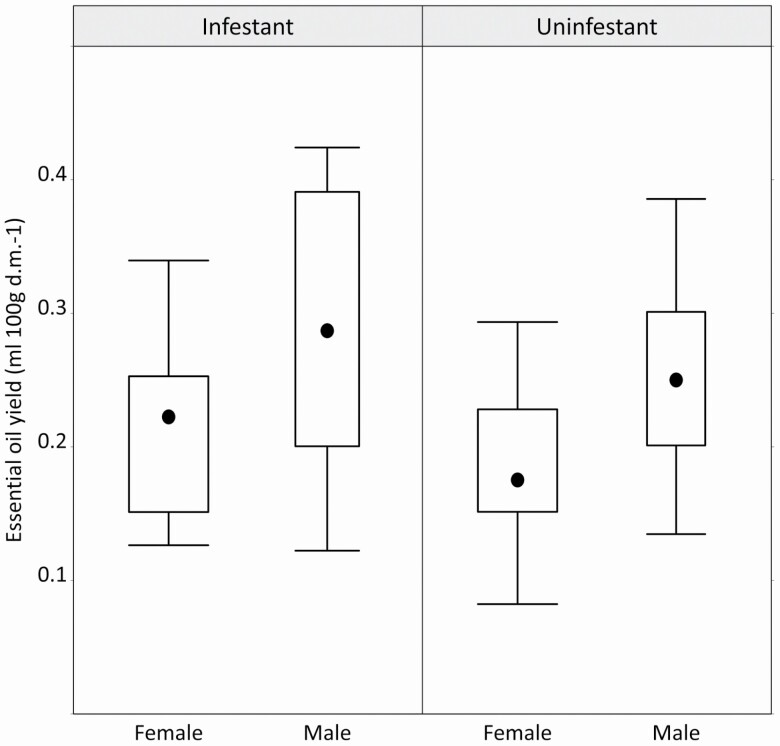
The Box-and-Whisker plot shows the essential oil yield values (expressed in mL/100 g dry mass) according to the presence of *Carulaspis juniperi* in each sex, separately. Variability is represented by the medians (black dot), interquartiles (boxes) and interquartile ranges (whiskers).

In the adult juniper shrubs, the essential oil yield significantly correlated with the sex, age, canopy position and scale insect infestation, but not the population and the interactions (Table 2). In particular, we found that sex-specific essential oil yield for males contained a much higher volume than for females, and the yield increased consistently within the canopy along a vertical gradient in both sexes (Fig. 1). We also detected that the essential oil yield decreased with age (Fig. 2), while the scale insect-infested individuals showed a higher essential oil yield than the insect-free shrubs (Fig. 3).

**Table 2.** GLMM analyses of the essential oil yield in adult junipers. GLMM analyses of the essential oil yield values in adult junipers are shown where the stars represent the significance level of the tested variables (*P < 0.05; **P < 0.01; ***P < 0.001).

Variable	*F*-value	d.f.	*P*-value
Sex	9.69	1, 42	0.003**
Infestation	4.87	1, 82	0.030*
Canopy position	10.82	2, 82	<0.001***
Age	8.71	1, 42	0.005**
Site	0.09	2, 42	0.909
Infestation × Canopy position	2.49	2, 82	0.088
Infestation × Sex	0.06	1, 82	0.795
Infestation × Age	0.63	1, 82	0.428
Sex × Canopy position	0.57	2, 82	0.567
Sex × Age	2.19	1, 42	0.145
Canopy position × Age	0.50	2, 82	0.604
Random factor			0.002**

We described the relationship between the essential oil yield and ageing in juvenile and adult shrubs by a bi-exponential function defined by the following model formula:


$$\begin{array}{l} y=e^{-0.0329x}-e^{-0.08564x}, \end{array}$$

where *x* represented the age and *y* the essential oil yield (Fig. 2). The young shrubs showed an increased accumulation of the essential oil yield with ageing. The accumulation turnover (i.e. maximum function peak) was predicted at around 18 years. Concordantly with previous analyses, the yield decreased with lifetime in adults and converged to zero in the senescent shrubs. However, available data supported the model prediction in the very late senescent interval much less.

### Sexual differences in the growth and growth-defence trade-off

**Figure 4.**
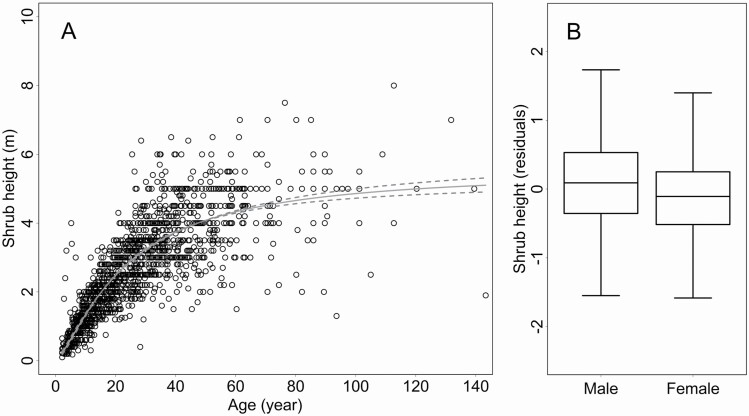
The plots show the growth patterns of the *Juniperus communis*. (A) The scatter plot shows the relationship of the shrub height with age approximated by a sigmoid curve (continuous line) with the 95 % confidence intervals (dashed lines). Each point represents a juniper individual. (B) The Whiskers-and-Box plot shows the sex-specific differences in the age-corrected shrub height.

We described a strong association between the height and the age of juniper shrubs which was approximated by a sigmoid curve (formula based on the full model:


$$\begin{array}{l} y=\frac{5.54}{1+\exp\left(\frac{3.15-\log(x)}{0.74}\right)}, \end{array}$$

where *x* represented the age and *y* the shrub height) to calculate the individual residuals for hypothesis testing (Fig. 4A). We found that male shrubs grew significantly higher (sex: *F* = 39.52; d.f. = 1, 1458.3; *P* < 0.0001) than females and this sex-specific difference was consistent throughout their lives in all populations (Fig. 4B).

**Figure 5.**
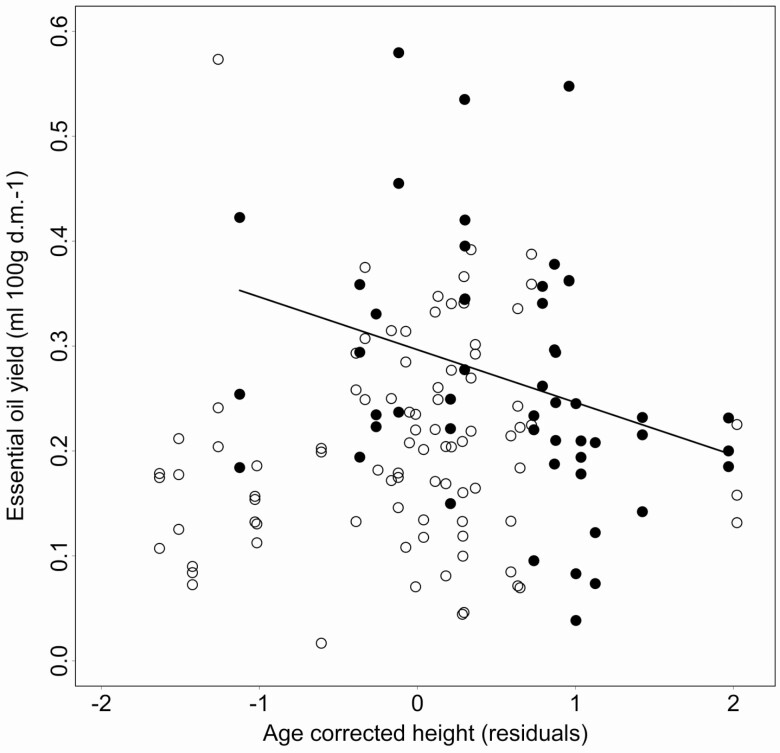
The scatter plot shows the sex-specific relationship between the essential oil yield values (expressed in mL/100 g dry mass) and the age-corrected shrub size (filled circle: male shrubs; open circle: female shrubs). The regression line represents a significant relationship in males.

We found another sexual dissimilarity in the relationship between the essential oil yield and the shrub size, reflecting the phenotypic manifestation of the growth-defence trade-off (Fig. 5; Table 3). The essential oil yield was correlated negatively with relative shrub size in males, while a similar trend was not detected in females.

**Table 3.** LMM analyses of the growth-defence trade-off in adult junipers. Table shows the statistical analysis related to the growth-defence trade-off in adult junipers. The stars represent the significance level of the tested variables (*P < 0.05; **P < 0.01).

Variable	*F*-value	d.f.	*P*-value
Size	4.47	1, 42.74	0.040*
Sex	10.94	1, 43.03	0.002**
Size × Sex	4.21	1, 42.75	0.046*

### Population sex ratio

**Figure 6.**
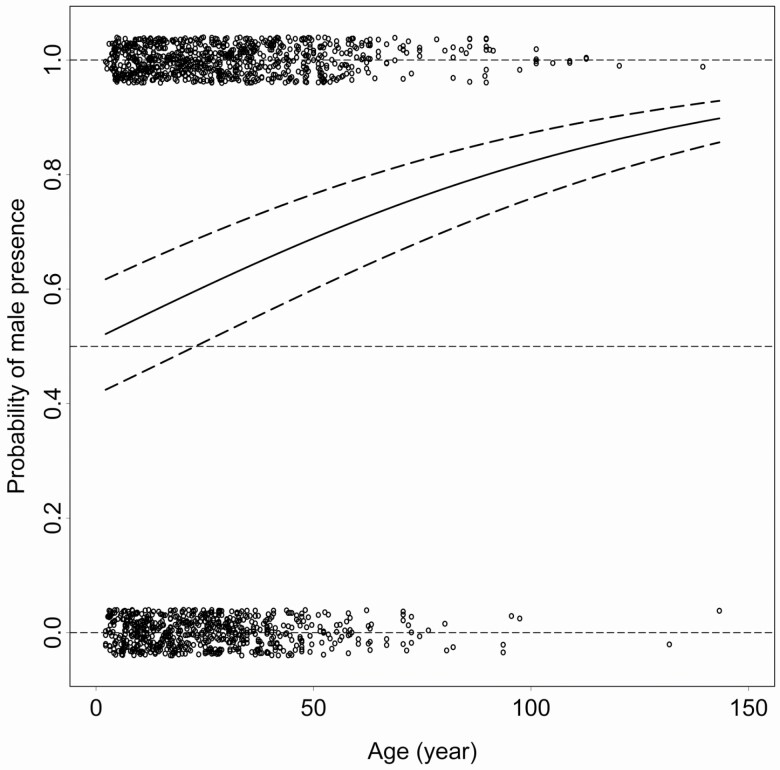
The plot shows the age-dependent frequency of the juniper individuals. Each point represents a binary value (0: females; 1: males) describe the individual sex. Values were slightly jittered for better visualization. The curve (continuous line) represents the probability of male presence (sex ratio) with ageing with the confidence intervals (dashed lines). The representative probability values, as total female dominance, equal sex ratio and total male dominance, were illustrated by the horizontal long dashed lines at 0, 0.5 and 1, respectively.

The relative frequency of the male juniper shrubs increased with age (age: χ ^2^ = 20.416, d.f. = 1, *P* < 0.0001, AIC = 2007.4; full model: AIC = 1985.4) resulting in a male-biased community structure especially in the senescent generations. However, whereas the higher male frequency was clearly observable in all populations (site: χ ^2^ = 4.1987, d.f. = 2, *P* = 0.122, AIC = 1989.2), the level of the sex ratio inconsistently changed with ageing among the observed populations (site × age interaction: χ ^2^ = 7.617, d.f. = 2, *P* = 0.022, AIC = 1989.0) (Fig. 6).

## Discussion

Several studies have reported allocation trade-offs among the functionally different metabolic processes, such as growth, chemical protection, and reproduction, due to the conflicting utilization of the common and limitedly available nutrient resources (e.g. Koricheva 2002; Züst and Agrawal 2017). Specifically, studies in dioecious species documented even more contrasted between-individual variances in phenotypic traits and biased sex ratio, reflecting the sex-specific nutrient allocation trade-offs due to the dissimilar reproduction investment (Ågren *et al.* 1999; Field *et al.* 2013a). Complementing the lacking or ambiguous knowledge in chemical ecology linked to dioecious conifers, the present study highlights the underlying growth-defence conflict mechanisms in *J. communis.*

We investigated the sex-specific dimorphisms in specific phenotypic traits, expecting differences in the essential oil yield, shrub size or both, assuming the existence of the growth-defence conflict. Supporting the general expectations (Ågren *et al.* 1999; Avila-Sakar and Romanow 2012), we detected consistent sex-specific differences in both the essential oil yield variation and growth ratio. However, despite such dimorphisms, we revealed a unique and contrasting pattern between males and females. Male junipers produced a higher yield of essential oil and grew consistently higher throughout their lives in all populations when compared with females; the former finding was the opposite, while the latter was in line with the general predictions of the growth-defence conflicts (Bañuelos *et al.* 2004; Cornelissen and Stiling 2005). Considering the sex-specific chemical and growth dimorphism, other juniper studies also documented that males tended to produce a higher yield of essential oil and invest more into growth than females (Adams and Powell 1976; Massei *et al.* 2006; Zheljazkov *et al.* 2013; Stark and Martz 2018). However, such pattern cannot be generalized across various taxa (Jing and Coley 1990; Marion and Houle 1996; Massei *et al.* 2006) and is often explained by interferences between physiological constraints and environmental stressors (e.g. Field *et al.* 2013a; Simpson 2013). Despite sex-specific allocation differences often described by the total yield of a PSM group, this robust analytical approach might be limitedly suitable for verifying detailed allocation trade-off structures. However, chemical polymorphisms could generate variance in chemical defence (e.g. Markó *et al.* 2011), suggesting more complex allocation patterns. Thus, future studies should focus more on the less known metabolite-specific aspects of the underlying mechanisms. Additionally, due to the structural and physiological reasons, the canopy position can be a potential driver affecting the chemical defence via the ontogenetic change in leaf quality (Lawrence *et al.* 2003) or the imbalanced light environment shaping the photosynthetic activity of the C-based PSMs productions (Herms and Mattson 1992). This was the first juniper study revealing high within-individual canopy differences in chemical defence, showing an increased yield along a vertical gradient in both sexes, suggesting subindividual variations in the chemical defence with different herbivore risk among tissues. Therefore, focussing on the subindividual patterns within the growth-defence conflict was found to be more relevant than we previously expected.

Our study supported the sex-specific existence of the growth-defence trade-offs in *J. communis* in line with the general expectations of the nutrient allocation trade-off conflicts (Koricheva 2002; Züst and Agrawal 2017). In spite of the strong sex differences in both phenotypic traits, we found that the essential oil yield was correlated negatively with relative shrub size but only in males. An allocation trade-off is most likely to appear under specific habitat- and taxon-specific conditions such as low resource availability and lack of herbivores, and inherently slow-growth type, producing metabolically expensive defence chemicals (Coley *et al.* 1985; Bazzaz *et al.* 1987). In contrast, such trade-offs might remain hidden in nutrient-rich habitats (Züst and Agrawal 2017). In general, our study system (i.e. habitat, species) fulfilled these conditions allowing us to detect allocation trade-offs in both sexes (Ward 1982; Kertész *et al.* 1993; Gershenzon 1994). However, these conditions could rarely occur simultaneously and permanently in natural systems, generating distinct allocation trade-off patterns. Therefore, these varying patterns suggest that physiological trade-offs between defence and growth could correlate more strictly in juniper males and more flexibly in females or caused by specific environmental conditions.

Such dissimilar or hidden sex-specific trade-offs highlight the role of phenotypic plasticity and environmental factors. The phenotypic plasticity can compensate for the suboptimal environmental fluctuations (Valladares *et al.* 2014), but the external environmental factors often interfere with allocation trade-off mechanisms (Koricheva 2002). Generally, a more adverse environment could generate higher physiological allocation trade-offs in both sexes, decreasing the sex-specific differences in dimorphism (DeSoto *et al.* 2016). Thus, similarly to our system, males and females could present different allocation strategy. For instance, both sexes could reduce their growth with the increase of unfavourable environmental conditions; however, males show higher tolerance to environmental challenges than females, while females reduce their reproduction efforts more flexibly than males (Ortiz *et al.* 2002). Females with higher phenotypic plasticity could compensate better for the differential investments between growth and reproduction to maintain a more balanced physiological and nutrient equilibrium, resulting in less obvious growth-defence trade-offs (Queenborough *et al.* 2007). Males with lower reproduction cost could generate a more likely trade-off between growth and defence due to maximizing their defence potential to abiotic stressors and decreasing growth (Hultine *et al.* 2016). Moreover, nutrient deficiency could generate different growth-defence trade-offs between sexes based on their nutrient requirements due to the differential life-history (i.e. growth ratio) and reproductive functions (reviewed in Sampedro *et al.* 2011; Dorken and Van Drunen 2018).

Specific studies highlighted the temporal consequences of the nutrient allocation trade-offs assuming that different physiological processes based on co-expression and temporal constraints result in yearly and seasonal fluctuation (i.e. masting years, flowering/growth period) in perennials (Cepeda-Cornejo and Dirzo 2010; Redmond *et al.* 2019). Similar to the masting pines, a juniper female could generate such fluctuations in allocation growth-defence trade-offs being evident in a masting year but undetected in an average year. We could not support this hypothesis due to unavailable long-term reproduction data, but a further study is warranted. Note that allocation trade-off could be more complicated in certain species (e.g. junipers) with long-lived fruits ripening multiple cone generations simultaneously.

Earlier studies also suggested seasonal variations in the strength of allocation trade-offs in junipers. For example, the most reduced sex-specific differences in essential oil composition overlapped with the intensive vegetative growth (including pollination) (Adams and Powell 1976). Another study found that females produced the highest essential oil yield in winter and the lowest in summer (Markó *et al.* 2008), proposing that drought with temporal extremes could cause a relatively high abiotic stress during intensive growth than in winter. Driven by a similar mechanism, an inverse pattern was found in boreal forests in which the cold winter extremes imposed a relatively higher level of abiotic stress than the summer (Stark and Martz 2018). Moreover, these examples emphasized the alternative defence functions against abiotic factors such as UV light, or drought resistance (Herms and Mattson 1992), generating seasonal variance in the strength of allocation trade-offs. In a more general context, the within-plant allocation trade-offs could be effectively hidden or absent, and arise only after the occurrence of a specific ecological factor, increasing the heterogeneity among populations (McKown *et al.* 2017). Moreover, a similar selection environment could raise similar growth-defence allocation trade-offs (Defossez *et al.* 2018). Therefore, adaptive processes (e.g. defence against abiotic stress regimes) could neutralize the sex-specific differences through the allocation trade-offs which often lead to local adaptations (Massei *et al.* 2006; DeSoto *et al.* 2016; Stark and Martz 2018; Vázquez-González *et al.* 2020).

We documented male-biased juniper populations in our study sites (Fig. 6), which reinforced the general expectations, namely, contrasting growth-defence trade-offs could generate unequal sex ratio via the sex-specific survival favouring the sex with relatively lower physiological cost and higher protection (Cornelissen and Stiling 2005; Avila-Sakar and Romanow 2012). A comparative study (Field *et al.* 2013a) revealed the primarily species-specific characteristics that could raise sex-ratio bias; a male-bias was more likely to evolve in long-lived trees producing fleshy fruits, while a female-bias occurred more frequently in herbs and shrubs with clonal reproduction. Moreover, specific ecological factors, such as xeric habitats, high altitude or latitude, could also initiate a male-biased population (Marion and Houle 1996; Ortiz *et al.* 2002; Queenborough *et al.* 2007; Field *et al.* 2013b). Our results concurred with the general predictions regarding ecological contexts and taxonomical characteristics and partially explained how other studies detected consistent female-biased sex ratio within the genus *Salix* (Maldonado-López *et al.* 2014; Jiang *et al.* 2016; Gouker *et al.* 2020). However, taxonomic differences also incorporate the differences in PSM production in terms of physiological origin and cost (e.g. high: terpenes, low: phenolics) and defence efficiency (Herms and Mattson 1992; Gershenzon 1994; Koricheva 2002). Such taxon-specific PSMs differences could increase variance in sex ratio through the allocation trade-offs. Therefore, an interspecific comparison of sex ratio could lead to misleading conclusions in the absence of performing phylogenetic control.

Moreover, the allocation trade-off patterns could also vary along lifetime (Züst and Agrawal 2017), which could shape the equilibrium of sex ratio via the age-dependent chemical defence. Accordingly, our results showed a continually increasing male dominance with ageing; therefore, intra- or interspecific comparisons of sex ratio should also consider the demographics of age, which could be highly varied among young and senescent populations. Therefore, our results suggested (see Figs 2 and 6) that the population non-equilibrium in the male-female ratio was risen with the increasing sex-specific difference in the reproduction cost by reshaping the growth-defence allocation trade-offs.

Recently, environmental stressors (e.g. aridity) became more frequent under global climate change. The climatic anomalies more likely affect dioecious plants due to the sexual dimorphism in resource acquisition and tolerance for abiotic stressors generating biased and spatially segregated populations (Hultine *et al.* 2016). The resulting male-biased populations could impose further ecological consequences, mostly affecting the survival of the community members depending on the female production as a primary food source (Cornelissen and Stiling 2005).

## Conclusions

The present study provides a unique insight into the growth-defence resource allocation conflicts in *J. communis*, detecting sex-specific phenotypic trait variations, growth-defence trade-offs and male-biased demographic structures. It shows that the allocation trade-offs can be driven by conflicting utilization due to the common and limitedly available nutrient resources. Furthermore, sexes could represent different allocation strategies flexibly reflecting the current ecological challenges that promote local adaptation processes. Subsequent studies should focus more on how dioecious species and their PSM quality could be influenced by biotic and abiotic stressors that initiate local adaptation, and the biased sex ratio to reshape the community structure. These processes are expected to increase considerably due to environmental stressors becoming more common under global climate change.

## Supporting Information

The following additional information is available in the online version of this article—

Figure S1. Habitat view of the Hungarian juniper shrublands.

plab021_suppl_Supplementary_Materials

## Data Availability

Supplementary data: Supplementary_File1_data.xlsx

## Notes

**Populations & Communities. Chief Editor:** Jean Burns
