# Supplementary material for: Sex-specific differences in Juniperus communis: essential oil yield, growth-defence conflict and population sex ratio
Source: AoB Plants. 2021 Apr 22;13(3):plab021. doi: 10.1093/aobpla/plab021 (PMC8192244; doi:10.1093/aobpla/plab021)
Supplement: plab021_suppl_Supplementary_Materials [file plab021_suppl_supplementary_materials.zip › plab021_suppl_MarkoG_suppl_files_20167AoBplants.docx]

Sex-specific differences in *Juniperus communis* L.: essential oil yield, growth-defence conflict and population sex-ratio

Gábor Markó^1,2*^, István Németh^3^, Veronika Gyuricza^4^, Vilmos Altbäcker^5^

^1^ Department of Plant Pathology, Institute of Plant Protection, Hungarian University of Agriculture and Life Sciences, Ménesi út 44, Budapest, H-1118, Hungary

^2^ Behavioural Ecology Group, Department of Systematic Zoology and Ecology, Eötvös Loránd University, Pázmány Péter sétány 1/C, Budapest, H-1117, Hungary

^3^ Biotech Biostatistics and Programming, Parexel International, Hermina út 17. Budapest, H-1146, Hungary

^4^ Department of Ethology, Institute of Biology, Eötvös Loránd University, Pázmány Péter sétány 1/C, Budapest, H-1117, Hungary

^5^ Department of Nature Conservation, Institute of Game Management and Nature Protection, Hungarian University of Agriculture and Life Sciences, Guba Sándor utca 40, Kaposvár, H-7400, Hungary

*Corresponding author: Markó, G (e-mail: marko.gabor3@gmail.com)


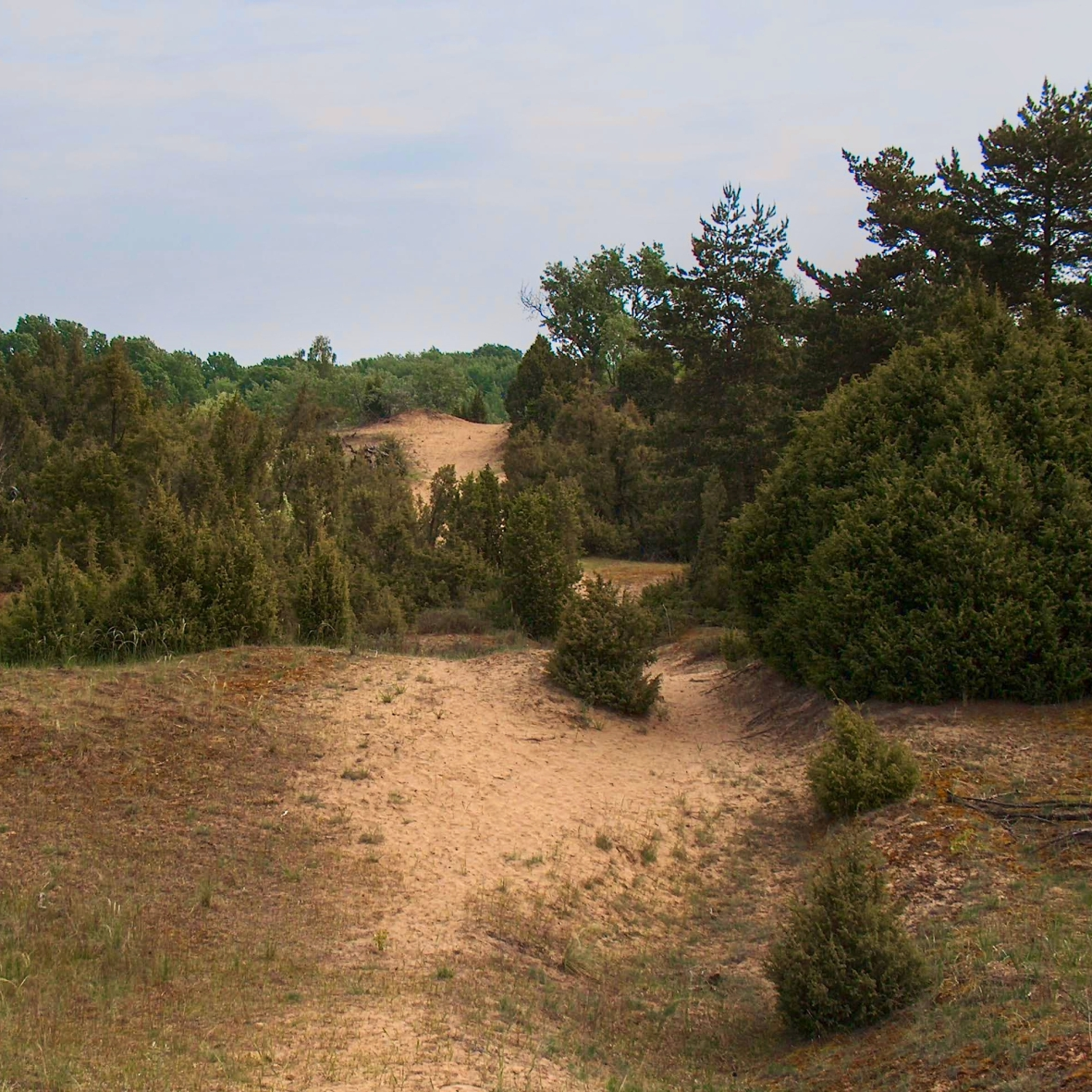


Juniper shrublands in the Kiskunság National Park (Hungary). Photo: Markó, G.


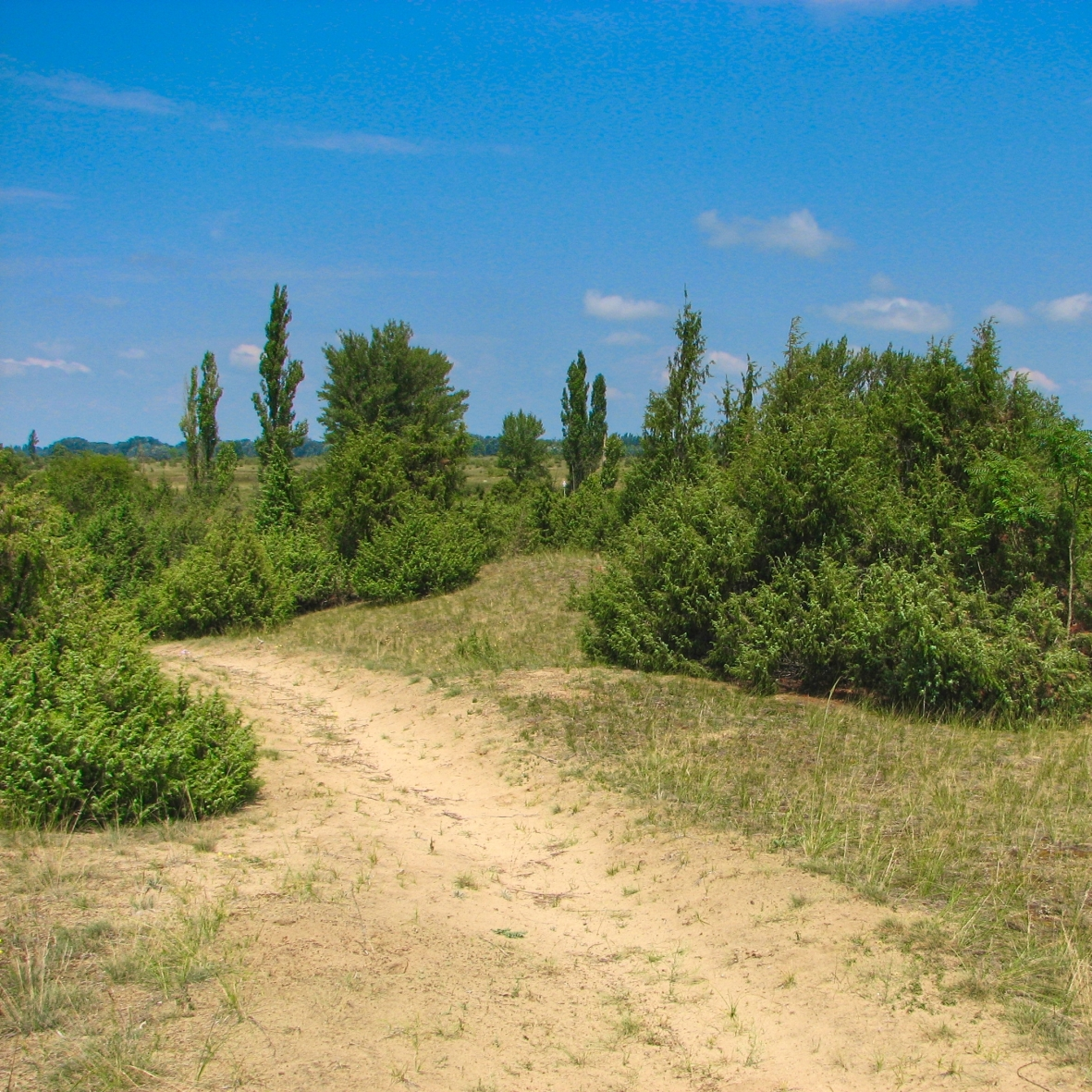


Juniper shrublands in the Kiskunság National Park (Hungary). Photo: Markó, G.
